# Understanding and Therapeutic Targeting of the p53 Network

**DOI:** 10.3390/cancers15184461

**Published:** 2023-09-07

**Authors:** Susanne Bacher, M. Lienhard Schmitz

**Affiliations:** Institute of Biochemistry, Medical Faculty, Justus-Liebig-University, Friedrichstrasse 24, 35392 Giessen, Germany; susanne.bacher@biochemie.med.uni-giessen.de

Signaling networks function as highly intertwined regulatory hubs rather than linear cascades with a single endpoint. The high degree of connectivity and regulation within networks through feed-forward and feedback loops introduces new features to the system, such as buffering, robustness, and reduction in biological noise [1,2]. This is particularly relevant for the p53 protein, not only due to its pathophysiological significance, but also because of its important contributions to major functions in human physiology. These include the regulation of cell proliferation and DNA repair, cell survival, metabolism and differentiation [3,4,5]. Although p53 is one of the most extensively studied proteins, our understanding of its role in physiology and cancer, where it is frequently mutated, remains incomplete. For example, there is good evidence that the tumor suppressor p53 opposes the metabolic changes commonly acquired during tumorigenesis, while other metabolic functions such as its role in controlling the levels of reactive oxygen species (ROS) do not reveal a coherent picture [6]. This limited understanding may also contribute to the fact that strategies aimed at reprogramming p53 function in cancer have achieved only modest clinical success so far.

Two review papers published in *Cancers* [7,8] now consider p53 from a different angle and discuss features of this tumor suppressor from the network viewpoint. This network comprises a number of proteins that interact with p53 in a functional or regulatory way. This novel perspective allows for previously potentially overlooked viewpoints, which can open up new experimental questions and therapeutic approaches.

The review by Lahalle and coworkers discusses not only key metabolic functions of wildtype (WT p53) and mutant p53, but also of other components of its regulatory network, namely the p53 ubiquitin E3 ligase MDM2 (Mouse Double Minute 2) and its partner MDM4, the multifunctional E4F1 protein, the tumor suppressor ARF (Alternative Reading Frame), and the polycomb complex protein BMI-1 (B lymphoma Mo-MLV insertion region 1 homolog) [8]. The inclusion of p53 network components for the control of metabolic processes may also help to explain incoherent findings, such as the aforementioned role of p53 for the control of ROS levels. For instance, chromatin-associated MDM2 contributes to glutathione (GSH) recycling and thus affects the cellular redox state [9]. Interestingly, this function also occurs in p53-deficient cells and thus allows p53-independent functions of the network components. On the other hand, the network also enables the modulation of p53-driven output programs, as genes for serine metabolism are regulated in opposite directions by chromatin-associated p53 and MDM2. Although the network components in tumor cells are not mutated as frequently as the p53 protein itself (≈50% of all human tumors), changes such as *MDM2* gene amplification or overexpression of the MDM2 protein are observed in liposarcomas [10]. These tumors show high levels of chromatin-bound MDM2, which presumably fuels serine metabolism to support nucleotide synthesis in these proliferating cancer cells. As Lahalle et al. point out, this chromatin function of MDM2 might explain the ineffectiveness of Nutlin-3A (a compound disrupting MDM2-p53 interaction) for the treatment of liposarcomas. Surprisingly, Nutlin-3A promotes the chromatin association of MDM2 in liposarcomas, thereby contributing to increased tumor-supportive nucleotide synthesis. Intriguingly, downregulation of MDM2 levels by the small molecule compound SP141 led to impaired growth of liposarcomas [11], showing that components of the p53 network can be suitable pharmacological targets.

The concept of the p53 network as a valuable resource for suitable targets is also at the center of the *Cancers* review by Brown and colleagues [7]. They provide a timely introduction to the classical strategies of p53 therapies, which ultimately aim for the restoration of endogenous p53 function. Early attempts used suitable viral vectors to express the WT p53 protein in tumor cells, but clinical success is not seen. The same limitations apply to compounds such as the above-mentioned Nutlin-3a, which binds to MDM2 and thus releases p53 from its inhibitor. A third strategy aims to reverse the conformational changes that typically occur for mutant p53, thus allowing the restoration of p53 function. Although compounds such as PRIMA-1 and its derivative APR-246 (Eprenetapopt) show promising results in vitro, a recent clinical phase III study revealed rather disappointing results. Attempts to correct the conformation of mutant p53 by administering the chaperone HSP90 inhibitor Ganetespib in order to reactivate the tumor suppressor function of p53 also remained unsuccessful in clinical trials. Given these limited therapeutic successes, Brown et al. now propose a different conceptional approach: rather than directly focusing on p53 alone, the dysfunctions arising from the network formed by mutant p53 could be utilized as a basis for treatment strategies [7]. The authors discuss that the combination of Ganetespib with MDM2 and MDMX inhibitors increases the efficacy of treatment and provide further examples for possible combinatorial approaches. We are thus in need of a smart targeting strategy to tackle the p53 network at several sites to achieve the desired clinical outcomes, while limiting potential side effects. This strategy also requires precise definitions of the core components and the extended networks for WT p53 and its clinically most common mutants in the future.
